# AI-augmented risk stratification in pediatric cutaneous lupus: opportunities for EHR-derived phenotyping and early intervention

**DOI:** 10.1097/MS9.0000000000005848

**Published:** 2026-10-28

**Authors:** Ayesha Tariq, Sabeen Tahir, Zohaib Hassan, Rejina Chhetri, Janu Chhetri, Marina Kc

**Affiliations:** a Punjab Medical College, Faisalabad, Punjab, Pakistan; b Federal Medical and Dental College, Islamabad, Pakistan; c Nepalgunj Medical College, Kohalpur, Banke, Nepal; d Department of Medicine, Parbat Provincial Hospital, Nepal

*To the Editor,*

Pediatric cutaneous lupus erythematosus (CLE) presents persistent challenges in recognition, severity assessment, and longitudinal monitoring. The Cutaneous Lupus Erythematosus Disease Area and Severity Index (CLASI) measures cutaneous activity and damage^[^1^]^, while lesion subtype informs the assessment of systemic disease^[^2^]^. Pediatric validation supports the CLASI’s reliability and validity, although the cited study included only 11 patients and did not establish longitudinal predictive performance^[^3^]^. The pediatric-specific research need is to identify children at risk of recurrent activity, accumulating damage, or new systemic involvement over their subsequent disease course. Conventional clinical assessment, serial CLASI scoring, laboratory monitoring, and multidisciplinary follow-up remain essential. Electronic health record (EHR)-derived phenotyping could complement these approaches by integrating information across encounters and specialties; its incremental predictive value over conventional assessment remains unproven.

Artificial intelligence (AI) in dermatology is increasingly moving beyond single-image classification toward multimodal clinical reasoning. Dermatologic diagnosis depends on morphology, distribution, symptoms, temporal evolution, medical history, histopathology, and treatment response^[^4^]^. Longitudinal clinical notes may also document recurrence, treatment adherence, and systemic symptoms. Extracting these features could make information already available to clinicians more consistently usable for longitudinal analysis rather than replacing clinical judgment or the CLASI.

Recent work in adult systemic lupus erythematosus (SLE) used natural language processing to characterize EHR-derived clinical phenotypes and disease trajectories^[^5^]^. A review describes broader machine-learning applications in SLE, including diagnosis, prognosis, biomarker discovery, and patient stratification^[^6^]^. These provide methodological precedents, not direct evidence of predictive accuracy or clinical benefit in pediatric CLE. The pediatric evidence cited here concerns CLASI validation^[^3^]^; none of the cited AI publications establishes a validated pediatric CLE risk-prediction model or improved outcomes from AI-guided care^[^4–6^]^. This gap warrants pediatric-specific model development and evaluation.

Four proposed prediction tasks should be distinguished. Recurrent flares could be studied using prior flare frequency, intervals of remission, lesion activity, and medication exposure. Inadequate treatment response could be assessed through serial activity scores, lesion persistence, treatment dose and duration, and documented adherence, separating insufficient exposure from apparent nonresponse. Progressive cutaneous disease could be defined by sustained worsening activity or new damage, using lesion distribution, scarring, alopecia, and serial CLASI activity and damage scores. New systemic involvement would require a separate, clinician-adjudicated outcome in children without established SLE, with candidate predictors including systemic symptoms, blood counts, complement levels, autoantibodies, and urinalysis. These are candidate features, not established predictors in pediatric CLE. Each task requires a pre-specified outcome and prediction horizon, using only information available before the predicted event to prevent data leakage.

AI-assisted risk stratification could be tested as a means of informing follow-up intensity, treatment review, or multidisciplinary assessment. These are potential applications requiring prospective validation, not demonstrations of clinical benefit. Such systems should be decision-support tools rather than autonomous diagnostic platforms. Clinical and, when indicated, histopathologic assessment remain necessary^[^4^]^; a model flag for systemic risk would prompt evaluation, not establish SLE. Studies should compare models with conventional assessment and simpler prediction methods, evaluating whether any added accuracy translates into useful decisions without excessive false alerts or unnecessary treatment.

Several challenges must be addressed before implementation. Pediatric CLE datasets are likely to be small and heterogeneous. Missing longitudinal data, inconsistent terminology, and class imbalance for uncommon outcomes could bias models. Differences in documentation and care practices may limit transportability, while temporal dataset shift requires reassessment as practice changes. External validation across pediatric centers, calibration of predicted risks, and explanations linked to clinically interpretable features are essential. Privacy protection, appropriate consent and assent, data governance, and evaluation of fairness across patient groups must accompany development.

Prospective, multicenter pediatric studies are therefore needed to determine whether EHR-derived phenotyping adds value beyond existing monitoring and whether its use can improve clinical outcomes. Until such evidence is available, AI-augmented risk stratification in pediatric CLE should remain a research proposal grounded in clearly defined outcomes and clinician oversight.

## Data Availability

No datasets were generated or analyzed during the preparation of this Letter to the Editor.

## Research registration unique identifying number (UIN)

Not applicable for Letter to the Editor as per journal.
